# The influence of obesity and fat distribution on ankle muscle coactivation during gait

**DOI:** 10.1371/journal.pone.0294692

**Published:** 2024-03-20

**Authors:** Wael Maktouf, Hamza Ferhi, Sébastien Boyas, Bruno Beaune, Sabri Gaied Chortane, Pierre Portero, Sylvain Durand

**Affiliations:** 1 Bioengineering, Tissues and Neuroplasticity, UR 7377, Université Paris-Est Créteil, Faculté de Médecine, Créteil, France; 2 Research Unit (UR17JS01) « Sport Performance, Health & Society », Higher Institute of Sport and Physical Education of Ksar Saîd, University of “La Manouba”, Tunis, Tunisia; 3 Department of Sport Sciences, Laboratory “Movement, Interactions, Performance” (UR 4334), Faculty of Sciences and Technologies, Le Mans University, Le Mans, France; Università degli Studi di Milano: Universita degli Studi di Milano, ITALY

## Abstract

**Background:**

Excessive body weight is associated with gait alterations. In none of previous studies, body fat distribution has been considered as a factor that could change gait parameters and induce different neuromuscular adaptations.

**Objective:**

This multicenter, analytical, and cross-sectional study aimed to investigate the influence of the body mass distribution on gait parameters and ankle muscle coactivation in obese individuals.

**Methods:**

Three distinct groups were included in the study: a non-obese control group (CG, n = 15, average age = 32.8 ± 6.5 years, BMI = 21.4 ± 2.2 kg/m^2^), an obese-android group characterized by a Waist to Hip Ratio (WHR) greater than 1 (OAG, n = 15, age = 32.4 ± 3.9 years, BMI = 41.4 ± 3.9 kg/m^2^, WHR = 1.2 ± 0.2), and an obese-gynoid group with a WHR less than 1 (OGG, n = 15, age = 35.4 ± 4.1 years, BMI = 40.0 ± 5.7 kg/m^2^, WHR = 0.82 ± 0.3). All participants walked on an instrumented gait analysis treadmill at their self-selected walking speed for one minute. Spatiotemporal parameters, walking cycle phases, vertical ground reaction force (GRFv) and center of pressure (CoP) velocity were sampled from the treadmill software. Electromyography (EMG) activity of the gastrocnemius medialis (GM), the soleus (SOL) and tibialis anterior (TA) were collected during walking and used to calculate coactivation indexes (CI) between ankle plantar and dorsal flexors (GM/TA and SOL/TA) for the different walking cycle phases.

**Results:**

Compared to OAG, OGG walked with shorter and larger strides, lower CoP velocity and GRFv. During the single support phase, SOL/TA coactivation was higher in OAG compared to OGG (p < .05). During the propulsion phase, SOL/TA coactivation was higher in OGG compared to OAG (p < .05).

**Conclusion:**

Gait parameters and ankle muscle coactivation in obese individuals seem to be strongly dependent on body mass distribution. From the biomechanical point of view, body mass distribution changes gait strategies in obese individuals inducing different neuromuscular adaptations during the single support and propulsion phases.

## Introduction

Obesity is a major public health concern, as it is known to increase the risk of dependency and limit mobility in adults [1]. Over the past decade, obesity rates have skyrocketed worldwide. The condition is associated with a high risk of developing walking limitations, which is particularly concerning given the importance of walking for autonomy, disease prevention, and weight management [2]. The excess body weight associated with obesity modifies body geometry by adding mass to different regions, which can have a significant impact on the biomechanics of activities of daily living [3]. As such, understanding the relationship between obesity, body weight distribution, and gait parameters is crucial for developing effective interventions to improve mobility and quality of life for obesity individuals.

Recent research has shed light on the effect of obesity on gait in adults [4, 5]. Studies have reported that obese adults exhibit alterations in their spatiotemporal and kinetic gait parameters, including lower preferred walking speed and stride length compared to adults with normal weight [5, 6]. Obese individuals also tend to walk with a wider stride and spend more time in the double support gait phase [4]. The observed changes in gait are likely due to the biomechanical consequences of excess weight, which can lead to altered joint loading and increased energy cost of locomotion [7]. Understanding these gait alterations is important for the development of effective interventions to improve mobility and quality of life in obese individuals.

When considering the localization of adipose tissue in the body, two main types of obesity are described: android obesity and gynoid obesity. In android obesity, fat is mostly localized in the upper part of the body, while in gynoid obesity, fat is preferentially accumulated on the thighs and buttocks. These two types of obesity may induce different modifications in the geometry of body segments, raising questions about whether and how body fat distribution influences gait patterns. In this context, Menegoni et al. [3] were the first to study the effect of body mass distribution on postural parameters during static balance. Their study involved comparing obese women with gynoid obesity to men with android obesity, and revealed that an increase in body mass tends to lead to anteroposterior instability in both genders and medial-lateral destabilization in men specifically. However, Cieślińska-Świder et al. [8] reported contradictory results, showing that obese individuals with android-type obesity might be exposed to a greater risk of postural instability compared to those with gynoid-type obesity. Considering these studies, some aspects of the relationship between postural stability and body fat distribution continue to elude complete clarity. Furthermore, the impact of body fat distribution on gait parameters remains predominantly unexplored. As walking is a notable prerequisite for autonomy and plays an important role in disease prevention and weight management, understanding the relationship between obesity, body fat distribution, and gait parameters is crucial for improving mobility and quality of life in individuals affected by obesity.

During the walking support phase, young adults produce a net moment at the ankle joint to stabilize and propel their body mass [9]. This force production is ensured by muscle coactivation, which is the simultaneous activation of agonist and antagonist ankle muscles [10]. Muscle coactivation is a neuromuscular mechanism that allows the agonist muscles to work fluently and to increase joint stabilization while walking [9, 11]. Nevertheless, high muscle coactivation may be associated with excessive energy expenditure and consequently with early fatigue [12, 13]. Moreover, excessive muscle coactivation increases postural rigidity and may restrict dynamic postural control [14]. In a recent study, we observed that obesity increases muscle coactivation of the soleus and tibialis anterior muscles around the ankle joint during static postural control [13]. Kim et al. [15] revealed that as weight increased, there was a corresponding increase in the rates of muscle activation, particularly in the tibialis anterior and soleus muscles. These findings suggest that heavier weight loads may increase activation of muscles that control ankle joints, potentially leading to muscle fatigue. This, in turn, can impair balance ability and increase the risk of falls. In another study, we reported that obesity was associated with an excessive gastrocnemius medialis activity during the propulsive phase of walking and a high activity of soleus and tibialis anterior during the single support phase [5]. However, there has been no study that has investigated ankle muscle coactivation during walking in obese adults or examined the impact of body fat distribution on this neuromuscular mechanism. Obtaining a better understanding of these factors could shed light on the underlying mechanisms that contribute to gait alterations in obese individuals and how these alterations are influenced by body fat distribution. This information would be valuable to clinical practitioners as it could help to specify the appropriate type of physical conditioning. With this goal in mind, we aim to evaluate the effect of obesity on ankle muscle coactivation during walking, and investigate how body mass distribution affects gait parameters and ankle muscle coactivation.

## Materials and methods

### Study design and recruitment of participants

**Fig 1:**
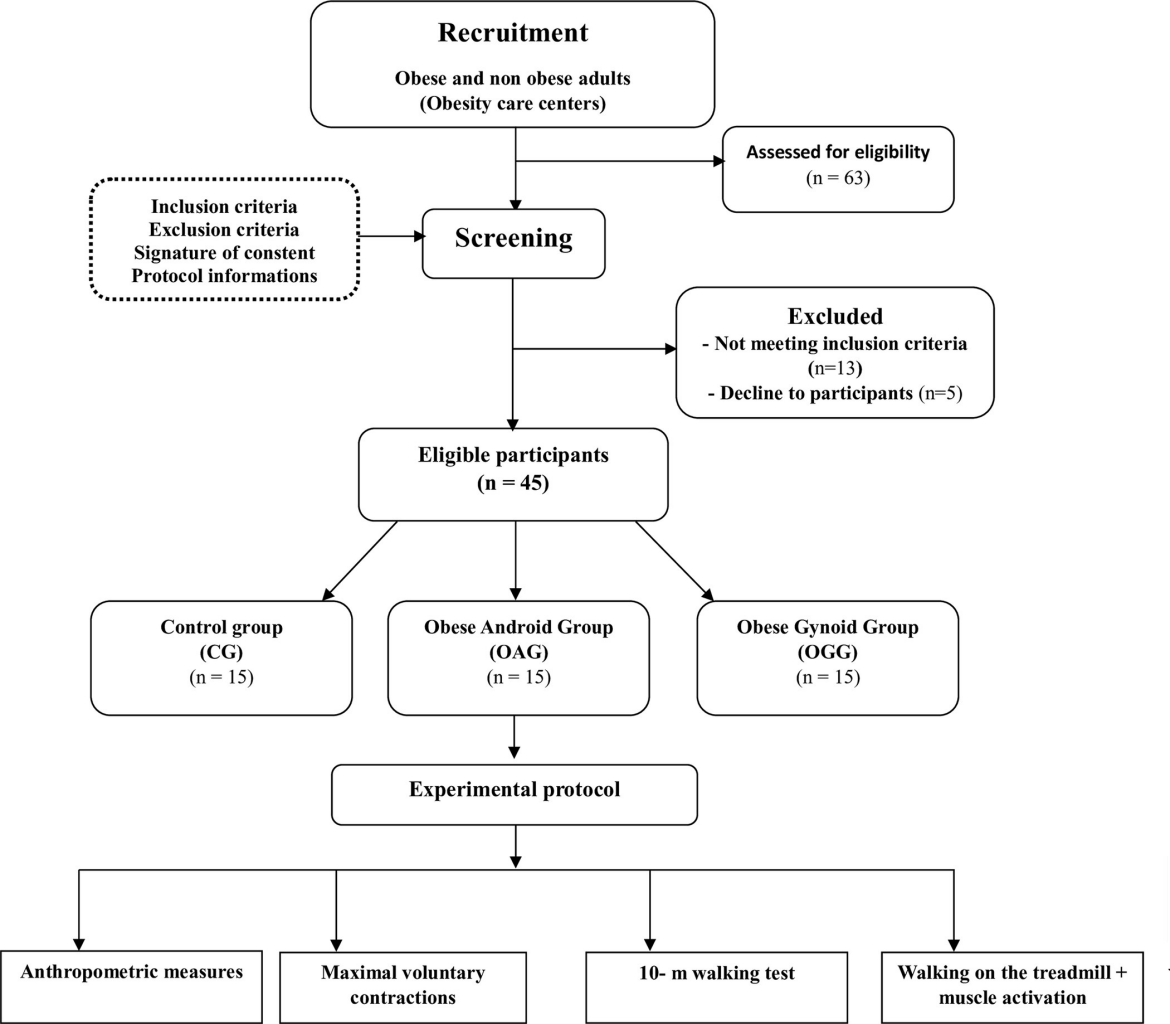
Flow diagram illustrating the experimental procedure design.

This study is a multicenter, analytical, and cross-sectional study (**Fig 1**). The study duration is 3 months, divided into three distinct periods: a recruitment period lasting 1–4 weeks, a screening period lasting 1–3 weeks, and a 17-week experimental testing phase. The entire experimental protocol is estimated to last between 60 and 75 minutes per participant. The first assessment involves taking anthropometric measurements to gather relevant data on participants’ body composition, including measurements such as height, weight, waist circumference, and body fat percentage. The second assessment involves a maximal voluntary contraction test of the plantar and dorsal flexors of the ankle. The third assessment is the walking test conducted across a 10-meter corridor. Lastly, the fourth test is the walk on the treadmill test.

The sample size was calculated using the freeware G*Power (version 3.1.9.4) [16]. The ANOVA test were predefined for power analysis. The estimation was based on predefined control of type I error (alpha = 0.05) and type II error (beta = 0.80), with moderate level of estimated effect size (f = 0.35). Under these setting, 45 participants were required as the minimum sample size. Participants for this study were recruited from various obesity care centers located across the region, between March and July 2022. A total of 63 individuals expressed interest in participating in the study. To ensure the study’s eligibility criteria were met, a thorough screening process was conducted. The inclusion criteria for this study were as follows: participants were required to be between 18 and 65 years old. For the non-obese control group (CG), a Body Mass Index (BMI, kg/m^2^) within the range of 18.5 kg/m^2^ to 24.9 kg/m^2^ was necessary. The obese android type group (OAG) required a BMI equal to or greater than 30 kg/m^2^, while the obese gynoid type group (OGG) had the same BMI requirement. Additionally, waist-to-hip ratio (WHR) criteria were used to further categorize participants [8]. Participants assigned to the android type group (OAG) needed to have a WHR exceeding 1, whereas those in the gynoid type group (OGG) had to have a WHR below 1. After applying these inclusion criteria, only 45 eligible participants were enrolled in the study, all of whom were female, and 95% of them were right-handed. These participants were then categorized into two distinct groups based on BMI: the control group (CG) and the obese group. Within the obese group, participants were further divided into two subgroups based on their WHR. The CG consisted of 15 normal-weight adults (age = 32.8 ± 6.5 years; BMI = 21.4 ± 2.2 kg/m^2^; WHR = 0.9 ± 0.3). The OAG included 15 obese adults with an android type (age = 32.4 ± 3.9 years; BMI = 41.4 ± 3.9 kg/m^2^; WHR = 1.2 ± 0.2), while the OGG consisted of 15 obese adults with a gynoid type (age = 35.4 ± 4.1 years; BMI = 40.0 ± 5.7 kg/m^2^; WHR = 0.82 ± 0.3). Individuals with a history of cardiovascular disorders, diabetes, uncorrected vision problems, severe musculoskeletal deformities, or injuries to their lower extremities that would interfere with testing were excluded from the study. The level of physical activity was assessed using the short version of the International Physical Activity Questionnaire [17]. All participants were informed about the study’s purpose and protocol and gave written consent. The study was conducted in accordance with the Declaration of Helsinki 2004 and was approved by the ethics committee on human research (C.P.P. SOUTH No. 0477/2022). Participant confidentiality and data privacy was strictly maintained throughout the study, and all data was analyzed in an aggregated and anonymous manner to ensure the anonymity of participants (Table 1).

**Table 1:** Participants’ physical characteristics.

Characteristics	CG (n = 15) Mean ± SD	OAG (n = 15) Mean ± SD	OGG (n = 15) Mean ± SD	ANOVA	
				F	*p*
Age (years)	32.8 ± 6.5	32.4 ± 3.9	35.4 ± 4.1	-	NS
Body height (cm)	169.0 ± 6.5	162.3 ± 5.8	162.3 ± 4.3	-	NS
Body mass (kg)	64.7 ± 5.9	109.0 ± 10.5*	105.4 ± 11.5*	89.7	p<0.001
BMI (kg/m^2^)	21.4 ± 2.2	41.4 ± 3.9*	40.0 ± 5.7*	110.7	p<0.001
Body fat (%)	13.8 ± 3.4	34.8 ± 6.7*	37.8 ± 7.2*	99.5	p<0.01
FBM (kg)	8.9 ± 2.9	37.9 ± 7.4*	39.8 ± 5.2*	91.9	p<0.001
LBM (kg)	55.8 ± 5.5	71.1 ± 5.1*	65.6 ± 5.1*	119.5	p<0.001
Waist circumference (cm)	71.4 ± 8.9	109.8 ± 6.0 *	94.8 ± 6.0 *+	87.5	p<0.001
Hip circumference (cm)	81.7 ± 6.7	95.4 ± 8.9*	115.4 ± 8.9*+	81.9	p<0.01
WHR	0.9 ± 0.3	1.2 ± 0.2	0.82 ± 0.3+	-	NS

CG: control group, OAG: obese group with android type, OGG: obese group with gynoid type, BMI: body mass index, FBM: fat body mass, LBM: lean body mass, WHR: the waist-to-hip ratio, SD: standard deviation.

NS: no significant difference.

* = Significant difference between CG and other groups (p < .05).

+ = Significant difference between OAG and OGG (p < .05).

### Anthropometric measurement

Participants’ body mass (BM) and height (H) were accurately measured using a digital floor scale and a wall-mounted stadiometer, respectively. This data was then used to compute the Body Mass Index (BMI) as:

$$BMI\;\left(\frac{kg}{m^{2}}\right)=BM\;(kg)/H^{2}(m)$$

Fat body mass (FBM, %) were measured using an impedance-meter (Tanita; SC 240-Class III; Tanita Europe B.V., Amsterdam, The Netherlands). FBM and lean body mass (LBM) were calculated as:

FBM=bodyfat(%)×BM;andLBM=bodymass–FBM

Waist circumference was measured at the midpoint between the last rib and the iliac crest, while hips circumference was measured at the level of the largest lateral extension of the hips. Both measurements were taken in a horizontal plane, as per the WHO STEPS protocol.

### Gait analysis

The participants’ preferred walking speed was assessed by conducting the 10-meter walk test [5] and subsequently recording their walking speed **(Table 2)**. Afterward, they were familiarized with walking on an instrumented gait analysis treadmill (Zebris FDM-T system; Zebris medical GmbH, Isny, Germany) at their pre-calculated preferred walking speed for 5 minutes, followed by a two-minute seated rest period. The participants were then asked to complete three one-minute trials, each separated by a five-minute seated rest period.

**Table 2:** Participants’ spatiotemporal parameters during walking.

	CG (n = 15) Mean ± SD	OAG (n = 15) Mean ± SD	OGG (n = 15) Mean ± SD	ANOVA			
				*F*	*p*	η2	*β*
Step length (cm)							
Left	65.5 ± 4.5	52.8 ± 4.4*	46.8 ± 4.1*+	417.3	p<0.001	0.80	1
Right	65.4 ± 4.3	53.4 ± 4.7*	45.4 ± 4.1*+	420.7	p < .001	0.81	1
Stride length (cm)	130.9 ± 11.5	106.2 ± 13.7*	92.2 ± 7.7*+	423.5	p < .001	0.81	1
Step width (cm)	8.6 ± 2.6	11.5 ± 2.5*	14.7 ± 3.7*+	116.4	p<0.001	0.52	1
Speed (m/s)	1.4 ± 0.2	1.0 ± 0.4*	0.7 ± 0.3*+	55.5	P<0.01	0.32	1
**Walking cycle**							
Support phase (%)							
Left	64.7 ± 1.9	66.8 ± 1.9*	66.6 ± 5.2*	40.5	p<0.01	0.27	1
Right	64.6 ± 1.9	67.3 ± 1.9*	67.3 ± 5.8*	48.5	p<0.01	0.29	1
1^st^ double support (%)							
Left	14.6 ± 2.0	16.9 ± 1.6	17.0 ± 2.0	-	NS	-	-
Right	14.7 ± 1.8	17.1 ± 1.8	17.2 ± 3.6	-	NS	-	-
Single support (%)							
Left	35.4 ± 1.9	32.7 ± 1.9*	32.6 ± 2.7*	40.5	p<0.01	0.28	1
Right	35.3 ± 1.9	33.2 ± 2.0*	33.0 ± 3.4*	41.2	p<0.01	0.26	1
2^nd^ double support (%)							
Left	14.8 ± 1.8	17.1 ± 1.8*	17.0 ± 4.3*	52.2	p<0.01	0.21	1
Right	14.6±2.0	17.0 ± 1.6*	17.1 ± 3.7*	51.7	p<0.01	0.22	1
Swing phase (%)							
Left	35.3 ± 1.9	33.2 ± 1.9*	33.4 ± 5.6*	20.1	p<0.05	0.17	1
Right	35.4 ± 2.0	32.7 ± 1.9*	32.7 ± 5.9*	20.5	p<0.05	0.16	1

CG: control group, OAG: obese group with android type, OGG: obese group with gynoid type, SD: standard deviation. NS: no significant difference

* = Significant difference between CG and other groups (p<0.05)

^+^ = Significant difference between OAG and OGG (p<0.05).

During data collection, gait parameters were recorded by the treadmill software (Zebris FDM software; Zebris medical GmbH, Isny, Germany) from the treadmill force plates at a sampling frequency of 100 Hz. Each trial lasted for 1 minute, but only data collected between the 10th and 50th seconds were considered to avoid any acceleration and deceleration effects in data collection [5]. Data collection included spatiotemporal parameters, walking cycle phases, vertical ground reaction force (GRFv), and Center of Pressure (CoP) displacements. The spatiotemporal parameters evaluated in this study were step length, step width, and stride length (measured in cm). The software categorized the walking cycle into two phases: the support phase (SU, %) and the swing phase (SW, %), further divided into 1st double support (1st DS, %), single support (SS, %), and 2nd double support (2nd DS, %). The absolute GRFv peaks were used to calculate peaks 1 and 2 of the relative GRFv (P1, P2, N/kg, respectively) by dividing the absolute value of GRFv peak by body mass. Additionally, CoP data was analyzed to determine the CoP length during the SU and SS (mm), the anteroposterior position of the CoP (measured in mm), and the CoP velocity (cm/s) during walking.

### Maximal voluntary contraction test

Maximal voluntary contractions (MVC) were recorded using a dynamometer (Sauter FL1K; Type: Force Gauge; Sauter GmbH, Balingen, Germany) while participants performed isometric contractions of the ankle plantar flexor (PF) and dorsal flexor (DF) muscles of their dominant leg [18]. Leg dominance was determined based on the preferred leg for kicking a ball, a reliable criterion for assessing inter-limb differences in unipedal postural control [19]. For PF, participants were instructed to maintain contact between their back, buttock, and thigh with the chair, stretch their leg horizontally, and exert force by pushing with the tips of their foot against the dynamometer. To ensure stability during PF contractions, a strap was used to firmly secure the dominant leg to the foot plane. In the case of DF, participants were asked to stand up, keep their ankle at a 90-degree angle, and push with their foot against the dynamometer [14]. Throughout each contraction, participants received strong verbal encouragement to ensure maximal effort. Two trials were conducted for each condition, with a 1-minute rest in between. The highest value from the two trials was used in the study.

### Electromyography recording

Electromyographic (EMG) data from the ankle joint muscles were recorded firstly during the MVC of DF and DF, and then, during the walk on the treadmill test using the Powerlab 16/35 system (Powerlab 16/35; sampling rate: 1000 Hz, ADInstruments, Dunedin, New Zealand) [5]. The EMG recording was time-synchronized with the treadmill data using a control device operating according to the principle of a synchronization switch system (ON/OFF). During the MVC and walking on treadmill tests, two unipodal surface electrodes (Uni-gel Single Electrode-T3425, Thought Technology Ltd., Montréal, Canada) were placed on three ankle muscles: the gastrocnemius medialis (GM), the soleus (SOL) and the tibialis anterior (TA) of the dominant leg. Before attaching the electrodes, the skin was carefully shaved and cleaned using an abrasive cleaner and alcohol swabs to reduce impedance. The placement and location of the surface electrodes, on the belly of each muscle, in parallel to muscle fibers orientation and with an inter-electrode interval of 20 mm, conformed to the recommendations of SENIAM.

The analysis of EMG data collected during the walk on the treadmill and MVC tests were post-processed using Matlab software (Matlab R2013a, MathWorks, Natick, USA), and the raw EMG signals were band-pass filtered at 15–500 Hz through a second-order Butterworth digital filter to remove noise or movement interference [20]. The data from strides between the 10th and 50^th^ of each walking trial were collected, and were rectified and smoothed using root mean square analysis (RMS) with a 20-ms window [21], calculated using the following equation [22]:

$$RMS_{\left(t\right)}=\surd \frac{1}{T}\;\int_{t0-T/2}^{t0+T/2}{\left(EMG\right)}^{2\;}dt,where\;T\;is\;the\;time\;of\;integration$$

For the MVC tests, a moving window with a width of 20 ms was used to find the maximum RMS EMG activity resulting from the three efforts of MVC for each kind of contraction. Then, all RMS EMG data of walking on the treadmill test were normalized to EMG MVC using the following equation for each muscle:

EMGRMS%[(RMSEMGduringwalking/RMSEMGMVC)x100%]

Then, the normalized RMS of the GM (RMS GM), SOL (RMS SOL) and TA (RMS TA) of each walking cycle’s phase were used in order to calculate the coactivation index using following equation [23]:

$$CI=\frac{2\;I\;antagonist}{I\;total}\times 100$$

where I antagonist is the area of the total antagonistic activity and I total is the integral of the sum of (EMG TA + EMG SOL) during the task, which were calculated using the following equations:

$$I\;\mathrm{antagonist}=\int_{t1}^{t2}RMS\;TA(t)dt+\int_{t2}^{t3}RMS\;GM\;(t)dt$$ (1)

Where t1 to t2 is the period in which the TA is working as an antagonist muscle (i.e., the RMS TA is less than RMS GM), t2 to t3 is the period during which the GM is working as an antagonist muscle, and I antagonist is the integral of the sum of these two periods.

$$I\;\mathrm{total}=\int_{t1}^{t3}(RMS\;agonist+RMS\;anatagonist)(t)dt$$ (2)

We noted that in these equations we used RMS GM and RMS TA in order to calculate the CI of (GM/TA). The same equations were used to calculate the CI of (SOL/TA), where RMS GM was replaced by RMS SOL.

### Statistical analysis

Statistical analyses were conducted using Statistica Software 13.0 (Software, Inc., Tulsa, USA). The normality of data distribution and homogeneity of variance were checked using the Kolmogorov–Smirnov and Levene tests, respectively. As the parametric assumptions were satisfied, a one-way ANOVA followed by Bonferroni post hoc tests were performed to compare the gait parameters and CI among the three groups (control, android type, and gynoid type). The results are presented as means (±95% confidence interval). A p-value of less than 0.05 was considered statistically significant. Partial eta-squared (η2) and study power (β) were also reported. In order to determine the magnitude of changes, the effect size (r) was calculated based on the values of *d* [24] from group-comparisons and significance. The effect size was characterized by Cohen [25] as weak, moderate and strong effects (*i*.*e*., *d = 0*.*2 is small*, *d = 0*.*5 medium and d = 0*.*8 large*, *respectively*).

## Results

### Gait parameters

The analysis of spatiotemporal parameters **(Table 2)** revealed significant differences between the control group (CG) and the obese groups. The obese participants demonstrated shorter step lengths and wider step widths compared to the control group (p< 0.05; r >0.8). Moreover, the step width was found to be significantly higher in the obese group with gynoid type (OGG) compared to the obese group with android type (OAG) (p< 0.05; r >0.7). Regarding the walking cycle phases, the support phase was shorter, and the swing phase was longer in the control group compared to the obese groups (p< 0.05; r >0.6).

The center of pressure (CoP) displacements during walking were influenced by obesity **(Table 3)**. The obese group with android type (OAG) exhibited higher CoP length and maximal velocity compared to the control group (p< 0.05; r> 0.8). Similarly, the obese group with gynoid type (OGG) showed significantly higher CoP length and maximal velocity compared to OAG (p< 0.05; r> 0.6). Analysis of the vertical ground reaction force parameters revealed differences between the two obese groups. During walking, the vertical ground reaction force (P1 and P2) was lower in the obese group with gynoid type (**Table 3**) compared to the obese group with android type (OAG) (p< 0.05; r> 0.6; p< 0.05; r> 0.6, respectively).

**Table 3:** Center of pressure and vertical ground reaction force parameters.

	CG (n = 15) Mean ± SD	OAG (n = 15) Mean ± SD	OGG (n = 15) Mean ± SD	Obesity effect			
				*F*	*p*	*α^2^*	*β*
**CoP parameters**							
Length in SU (mm)							
Left	161.1 ± 21.3	194.7 ± 12.4	170.9 ± 18.2*+	34.6	p<0.001	0.44	1
Right	161.4 ± 15.1	*192.5 ± 13.8	172.1 ± 20.5*+	32.6	p<0.001	0.49	1
Length in SS (mm)		*					
Left	113.5 ± 15.3	97.5 ± 24.6*	87.7 ± 19.5*	34.6	p<0.001	0.24	1
Right	113.8 ± 13.3	97.2 ± 24.4*	89.6 ± 22.2*	159.5	p<0.001	0.60	1
Anteroposterior position (mm)	169.1 ± 14.5	150.2 ± 17.6	138.5 ± 35.0*+	19.6	p<0.001	0.15	1
Maximal velocity (cm/s)	65.5 ± 15.5	107.7 ± 31.2*	87.8 ± 17.2*+	57.9	p<0.001	0.34	1
**GRFv parameters**							
Relative P1 (N/kg)							
Left	10.4 ± 1.1	10.2 ± 1.2	9.1 ± 1.3+	10.3	p<0.05	0.10	0.75
Right	10.2 ± 1.0	10.3 ± 1.2	9.2 ± 1.7+	11.5	p<0.05	0.11	0.76
Relative P2 (N/kg)							
Left	11.3 ± 2.7	10.5 ± 1.2	9.3 ± 2.1+	10.3	p<0.05	0.10	7.3
Right	11.7 ± 2.5	10.2 ± 1.6	9.2 ± 2.2+	10.5	p<0.05	0.11	7.5

CG: control group, OAG: obese group with android type, OGG: obese group with gynoid type, SD: standard deviation. NS: no significant difference

* = Significant difference between CG and other groups (p < 0.05)

^+^ = Significant difference between OAG and OGG (p < 0.05).

### Coactivation index

**Fig 2:**
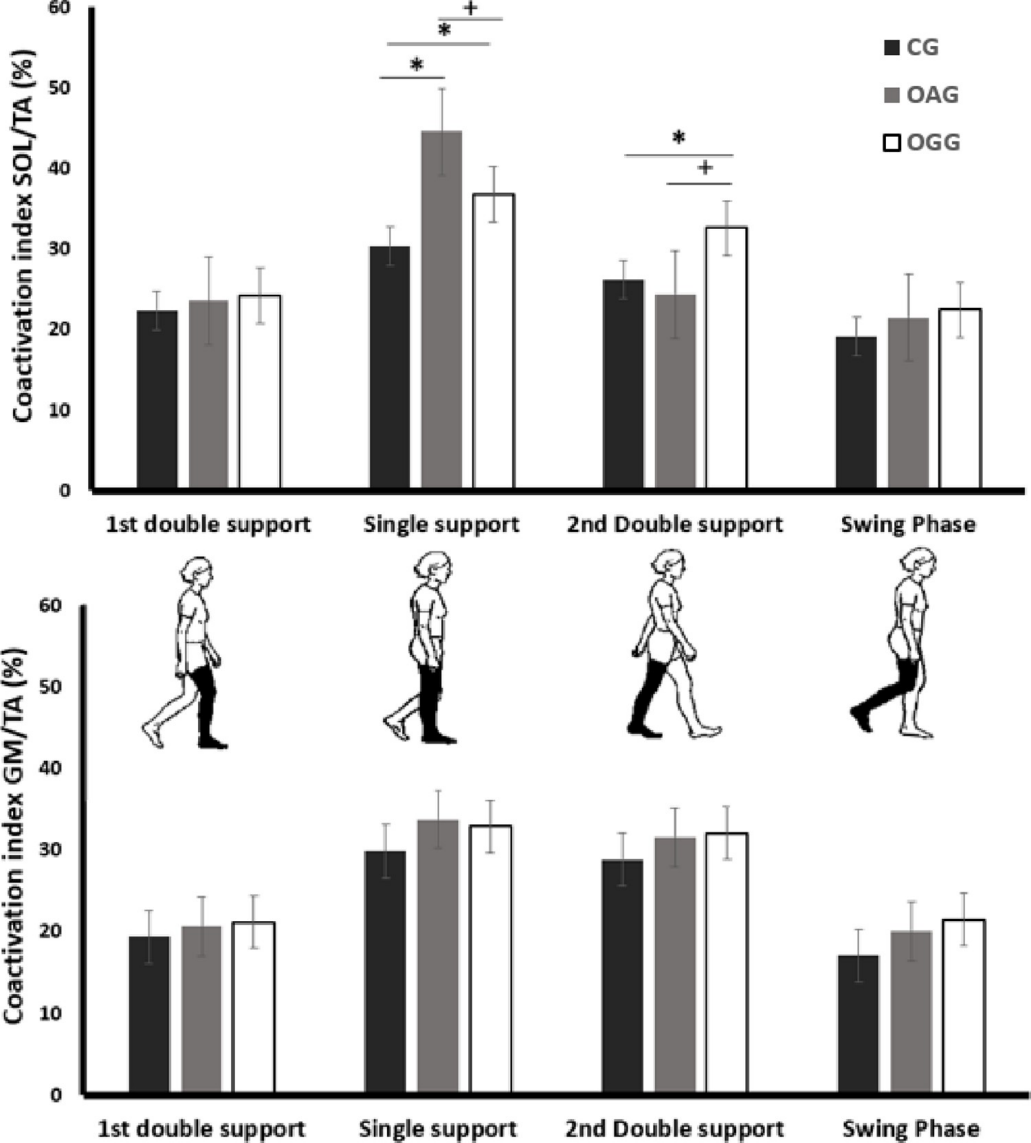
Coactivation index of ankle muscle joint during walking cycle phases. CG: control group, OAG: obese group with android type, OGG: obese group with gynoid type, * = Significant difference between CG and other groups (p <0.05). + = Significant difference between OAG and OGG (p <0.05).

The results of the SOT/TA coactivation analysis are presented in **Fig 2**. During single support and 2nd double support phases, SOT/TA coactivation was significantly higher in both OAG and OGG groups compared to the CG (p< 0.05; r> 0.8). Additionally, SOT/TA coactivation was significantly higher in OAG compared to OGG during the single support phase (p< 0.05; r>0.6). Conversely, during the 2nd double support phase, SOT/TA coactivation was higher in OGG compared to OAG (p< 0.05; r> 0.5).

## Discussion

This study yielded two major findings. Firstly, gait parameters are affected by excessive body fat mass, and muscle coactivation during the support phase is increased as a result. Secondly, body fat mass distribution causes changes in gait strategies among obese individuals, leading to different neuromuscular adaptations during the single support and propulsion phases.

### Effect of excessive body mass on gait parameters and ankle muscle coactivation

The negative impact of obesity on gait kinetics has been extensively documented in the literature [26, 27]. Our study results confirm these findings, indicating that obese individuals have lower step lengths and higher step widths, which alters the geometry of gait. These alterations in gait patterns are likely due to poor muscle performance, high metabolic expenditure, and physical exhaustion [7, 26, 28]. The present study’s significant finding is the high ankle muscle coactivation during the propulsion phase (+25% in OGG) in obese groups. A recent study suggested that body weight is a strong predictive factor of high gastrocnemius muscle activity during the propulsion phase [5]. The adaptive neuromuscular responses of ankle muscles may be due to the biomechanical modifications of gait associated with the mobilization of high body mass. For instance, obese individuals tend to decrease their knee flexion by 12% and increase their plantar flexion by 11% when walking at a comfortable speed, favoring an ankle strategy that increases stimulation of the plantar flexors during the propulsion phase [29]. This adjustment leans towards an ankle-centric strategy, intensifying the engagement of plantar flexor muscles during the propulsion phase [30]. This alteration in gait strategy is intricately linked to the unique biomechanics of the gastrocnemius muscle, a versatile biarticular muscle that exhibits remarkable adaptability [30]. When faced with varying mechanical demands, the gastrocnemius adeptly fine-tunes its activity. For instance, when the knee is flexed, the gastrocnemius serves as a potent knee flexor [31, 32]. However, in situations requiring greater force at the ankle joint, the simultaneous activation of the vastus muscles comes into play. This coordinated effort not only stabilizes the knee joint but also redirects the force generated by the gastrocnemius towards the ankle [33]. In light of these insights, we propose that the observed heightened coactivation of ankle muscles in our study signifies an adaptive and strengthening strategy. This strategy serves a dual purpose: firstly, it redistributes the load across the joints, potentially mitigating any discomfort or pain, and secondly, it ensures stability when confronting the dynamic challenges of walking [30]. The intricate interplay among modified gait patterns, joint kinetics, and neuromuscular adaptations emphasizes the particular relevance of further investigations, especially concerning obese individuals and their body fat distribution.

In the current study, we observed a significant increase in ankle muscle coactivation during the single support phase (+47% for OAG and +21% for OGG), as well as higher CoP velocity during walking in obese groups compared to the control group. These findings indicate that obese individuals rely on increased ankle muscle coactivation as a neuromuscular strategy to reduce dynamic postural control instability, particularly during unipodale position. Consequently, high muscle coactivation may be considered a mechanism that stiffens lower limb joints [34]. While these neuromuscular adaptations help counteract mechanical gait modifications, excessive muscle coactivation can decrease postural rigidity [35] and restrict the degree of freedom of dynamic postural control regulation [36]. Furthermore, the high energetic cost of increased muscle coactivation [37] can lead to early fatigue [38], which may impair the ability to produce the required force during walking and limit the ability to respond to postural perturbations [13, 39]. Despite postural control adaptation being able to reduce the effects of added weight and fatigue to some extent, it was still insufficient to fully compensate for these changes [38]. This may be due to the fact that fatigued muscles can undergo alterations in contractile efficiency, which can impair their ability to produce high-frequency responses to balance perturbations [40]. Consequently, while a high level of ankle muscle coactivation is a necessary adaptation during walking in obese individuals, walking could be particularly challenging for obese and this could ultimately constitute a risk factor of falls.

### Effect of body fat distribution on gait parameters and ankle muscle coactivation

Clinical practice distinguishes between two types of obesity that significantly differ in terms of the distribution of body fat mass [8]. This study results show that obese individuals with gynoid type walk with higher step width (+28%) and lower step length (-12%) than obese individuals with android type. This finding confirms that the distribution of body fat mass is an important factor affecting static postural control [8] and gait parameters. Gynoid obesity type fat distribution may increase the net metabolic cost of walking, which is significantly related to a great mediolateral displacement of CoP and greater step width [6]. Walking is characterized by an inverted pendulum mechanism of energy interchange [41]. During the transition from one inverted pendulum arc to the next one, a part of the expended energy is lost, requiring external mechanical work to accelerate the CoP in the three planes of movement. Thus, having a high step width could increase the loss of energy and require more external mechanical work [6]. This could explain the relevant gait alterations observed in individuals with gynoid obesity compared to those with android obesity.

Furthermore, android type fat distribution increases SOL and TA coactivation in individuals during the single support phase (+21%) compared to those with gynoid type. During the propulsion phase, ankle muscle coactivation is greater in obese individuals with gynoid type (+23%). These findings are important as they indicate that body fat distribution induces different neuromuscular adaptations during walking. Moreover, as observed in the two obese groups, gait alterations affect neuromuscular strategies, but this influence is not proportional to the degree of alteration. There are several possible explanations for the increased ankle muscle coactivation in obese individuals with android and gynoid obesity types. First, the high level of ankle muscle coactivation observed in individuals with android obesity during the single support phase could be explained by failed mechanisms of postural control regulation. In this context, Cieślińska-Świder et al. [8] confirmed that obese women with abdominal fat location showed less stability in their standing posture than women with fat localized on the thighs and buttocks. Indeed, the accumulation of abdominal fat causes a forward shift in the CoP, typically assumed to be relative to the ankle joint in an inverted pendulum model [42, 43]. This forward CoP displacement results in an increased gravitational torque that accelerates the body [42, 44]. Consequently, obese individuals are required to generate ankle torque more rapidly and with a significantly higher rate of force development to counteract this gravitational torque and this can lead to a reduction in postural control stability [18].

During walking, SOL and TA are the main stabilizing muscles of the ankle during the single support gait phase [45]. There is evidence that obese individuals with android type increase ankle muscle coactivation around the ankle joint to reduce instability during the unipodale position in response to the anterior position of CoP [42]. Second, the high level of coactivation in individuals with gynoid obesity type during the propulsion phase could be explained by the biomechanical modifications of gait. The central nervous system controls stability by adjusting mediolateral foot placement, but this potentially has a metabolic cost [46]. Walking with a high step width, as individuals with gynoid obesity type do, could increase mediolateral instability [6]. As explained above, the high step width could increase the loss of energy and require more external mechanical work, reflected in the high level of ankle muscle coactivation.

The results of this study suggest that body fat mass distribution plays a significant role in altering gait parameters and inducing different neuromuscular adaptations in obese individuals. The changes in gait strategies observed in obese individuals with android and gynoid fat distribution may increase the risk of musculoskeletal disorders and falls [1], as they tend to walk with higher ankle muscle coactivation during gait phases. Therefore, healthcare professionals should consider body fat mass distribution as a potential factor that could increase the risk of musculoskeletal disorders and falls in obese individuals and develop appropriate interventions to address this issue.

### Limits and perspectives

Previous research has shown that ankle muscle coactivation and ankle joint moment are closely related. An increase in ankle muscle coactivation during gait is generally associated with an increase in ankle joint moment. Thus, the results of our study showing increased ankle muscle coactivation in the obese group, particularly in the single support and propulsion phases, suggest that they may also experience increased ankle joint moments. Future research incorporating measures of ankle joint moments would be necessary to confirm this hypothesis. Additionally, it is important to consider other potential factors that may affect ankle biomechanics, such as foot structure, which was not measured in our study. Thus, the interpretation of our results should be made with caution, and further research is warranted to fully understand the relationship between ankle muscle coactivation, ankle joint moment, and potential musculoskeletal disorders or risk of falling in obese individuals.

## Conclusion

Gait parameters and ankle muscle coactivation in obese individuals seem to be strongly dependent on body mass distribution. From the biomechanical point of view, body fat mass distribution changes gait strategies in obese individuals, inducing different neuromuscular adaptations during the single support and propulsion phases. Clinical practitioners should take into consideration these different neuromuscular and gait modifications induced by body mass distribution to provide a more specific type of physical conditioning.
